# The Impact of Optic Nerve Movement on Intracranial Radiation Treatment

**DOI:** 10.3389/fonc.2022.803329

**Published:** 2022-02-24

**Authors:** Kun Qing, Ke Nie, Bo Liu, Xue Feng, James R. Stone, Taoran Cui, Yin Zhang, Jiahua Zhu, Quan Chen, Xiao Wang, Li Zhao, Shreel Parikh, John P. Mugler, Sung Kim, Joseph Weiner, Ning Yue, Anupama Chundury

**Affiliations:** ^1^ Department of Radiation Oncology, Rutgers-Cancer Institute of New Jersey, Rutgers-Robert Wood Johnson Medical School, New Brunswick, NJ, United States; ^2^ Department of Radiology, University of Virginia, Charlottesviile, VA, United States; ^3^ Department of Radiation Oncology, City of Hope Medical Center, Duarte, CA, United States; ^4^ Department of Radiation Oncology, University of Kentucky, Lexington, KY, United States; ^5^ Department of Biomedical Engineering, Zhejiang University, Hangzhou, China; ^6^ Department of Medicine, Tuoro School of Osteopathic Medicine, New York, NY, United States

**Keywords:** radiation-induced optic neuropathy, MRI, optical nerve, stereotactic radiodiotherapy, radiation therapy

## Abstract

**Purpose:**

In radiotherapy, high radiation exposure to optic nerve (ON) can cause optic neuropathy or vision loss. In this study, we evaluated the pattern and extent of the ON movement using MRI, and investigated the potential dosimetric effect of this movement on radiotherapy.

**Methods:**

MRI was performed in multiple planes in 5 human subjects without optic pathway abnormalities to determine optic nerve motion in different scenarios. The subjects were requested to gaze toward five directions during MRI acquisitions, including neutral (straight forward), left/right (horizontal movement), and up/down (vertical movement). Subsequently, the measured displacement was applied to patients with peri-optic tumors to evaluate the potential dosimetric effect of this motion.

**Results:**

The motion of ON followed a nearly conical shape. By average, the anterior end of ONs moved with 10.8 ± 2.2 mm horizontally and 9.3 ± 0.8 mm vertically, while posterior end has negligible displacement. For patients who underwent stereotactic radiotherapy to a peri-optic tumors, the movement of ON in this measured range introduced non-negligible dosimetric effect.

**Conclusion:**

The range of motion of the anterior portions of the optic nerves is on the order of centimeters, which may need to be considered with extra attention during radiation therapy in treating peri-optic lesions.

## Introduction

Radiation-induced optic neuropathy (RION) can be a severe ocular complication in patients treated with radiotherapy (1). It often presents as an acute, profound, and irreversible loss of vision about 10-20 months after radiation exposure (2). It is believed that necrosis resulting from an overdose of radiation to the anterior visual pathway (AVP) is the primary cause of RION. Dosimetric studies have demonstrated that RION can occur when the cumulative dose to the AVP exceeds 50 Gy for conventional fractionation, or beyond 10Gy in a single fraction (2).

During radiation treatment, a planning margin, either a planning target volume (PTV) relative to clinical target volume (CTV) or planning risk volume (PRV) relative to organs at risk (OAR), is typically given to compensate for inter-/intrafractional motion and setup uncertainties. A 3-5 mm expansion is used for the optic apparatus in conventional radiation treatment, and a 0-1 mm expansion is used for single-/hypofractionated stereotactic radiosurgery (SRS). This concept is particularly crucial in SRS treatments, as the sharp dose falloff required near critical structures necessitates the reduction of small positional uncertainties that could have a sizable effect on radiation dose.

Compared to the optic chiasm (3), the range of movement for the optic nerve is known to be higher (4). Furthermore, instructing patients to fix their eyes on a single point for 5 minutes, or to close their eyes, did not significantly reduce movement (5). These findings implied the occurrence of non-negligible movement of the optic nerve, which may affect the dose exposure during radiotherapy. An accurate understanding of the magnitude of optic nerve movement is fundamental to the development of intracranial radiation treatment plans that minimize the risk of RION. The purpose of this study is to investigate the pattern and extent of optic nerve motion using serial MRI scans, and evaluate the potential dosimetric influence of this motion in representative patients with peri-optic tumors.

## Methods

### Human Subjects and Image Acquisition

The MRI study was approved by the Institutional Review Board at University of Virginia. Five healthy subjects without any orbital or optic apparatus abnormalities were recruited, including three young subjects (ages 24, 30, and 35 yrs., all males) and two older subjects (a 65-year-old female and a 67-year-old male). MRIs were performed on a 3T MR scanner (Siemens Magnetom Prisma, Erlangen, Germany) with a 64-channel head/neck coil. Images were taken with T2-weighted FLAIR-HASTE acquisition (duration: 50-74 seconds, resolution: 0.7 x 0.7 x 3.0 mm^3^, slice number 8-12 slices, TE/TR=90/3060ms, GRAPPA factor=4). All subjects were scanned in the supine position and were instructed to gaze in five different directions during MRI acquisitions: neutral (straight forward), left/right (horizontal movement), and up/down (vertical movement). Axial plane images were acquired for both eyes to assess horizontal motion and sagittal plane images of the right eyes were obtained to evaluate vertical motion. To be able to reproduce eye motion, extra scans assessing horizontal movement were taken on two different days within a 14-day window for the three younger subjects.

### Motion Assessment

On day 1, a baseline was established using the acquired MRI with the eyes in the neutral position. All other scans captured at different eye positions or on different days were rigidly aligned to the baseline using Velocity (Varian Medical Systems, Palo Alto, CA). Three measurement points were created, an anterior end connecting to the globe, a posterior end connecting to the optic canal along each optic nerve, and a middle point with equal distance between the optic nerve and the anterior/posterior ends. The L-R displacement was calculated along the axial plane to represent the horizontal movement of the eyes, while the S-I motion was assessed along the sagittal plane to represent vertical movement. The reproducibility of these points was also evaluated by comparing the repeated results to the initial ones. The extent of movement was further determined from the average displacement of the optic nerve as measured in the all subjects. Finally, a new motion-inclusive model was constructed based on the pattern of physiological movement of the optic nerve established from this study using Solidworks (Dassault Systèmes, Velizy-Villacoublay).

### Dosimetric Impact

To evaluate the potential dosimetric effect of optic nerve movement, three patients with peri-optic tumors (defined as a lesion within 3-5 mm of the optic nerve) were identified: Patient #1 was diagnosed with a meningioma that was treated with external beam radiation utilizing two partial arcs to a total dose of 6600 cGy in 33 fractions on a Truebeam (Varian Medical Systems, Palo Alto, CA); Patient #2, was diagnosed with a pituitary adenoma that was treated with hypofractionated stereotactic radiosurgery to a total dose of 2500 cGy in 5 fractions using the Gamma Knife Icon™ (Elekta, Stockholm, Sweden); and Patient #3, who was diagnosed with an optic nerve sheath meningioma treated with proton beam therapy to a total dose of 5220cGy RBE in 29 fractions with the Mevion S250 Proton Therapy System (Mevion Medical Systems, Littleton, MA).

The planning images (CT or MRI), plan dose, and contours were transferred to Eclipse (Varian Medical Systems, Palo Alto, CA) for direct comparison. We then further simulated the effect of optic nerve movement by creating a new estimated contour that was conically expanded from the original contour, effectively generating a motion-inclusive margin. The maximum point dose (D_max_ or D_0.035cc_) and D_0.2cc_ to the optic nerve on both the conventional and motion-inclusive margin models were obtained, while the D_min_ to the GTV and D_95_ for the PTV were reported in the worst-case scenarios and then compared with the actual/original values.

## Results

### Representative Images Showing Movement Pattern

**Figure 1:**
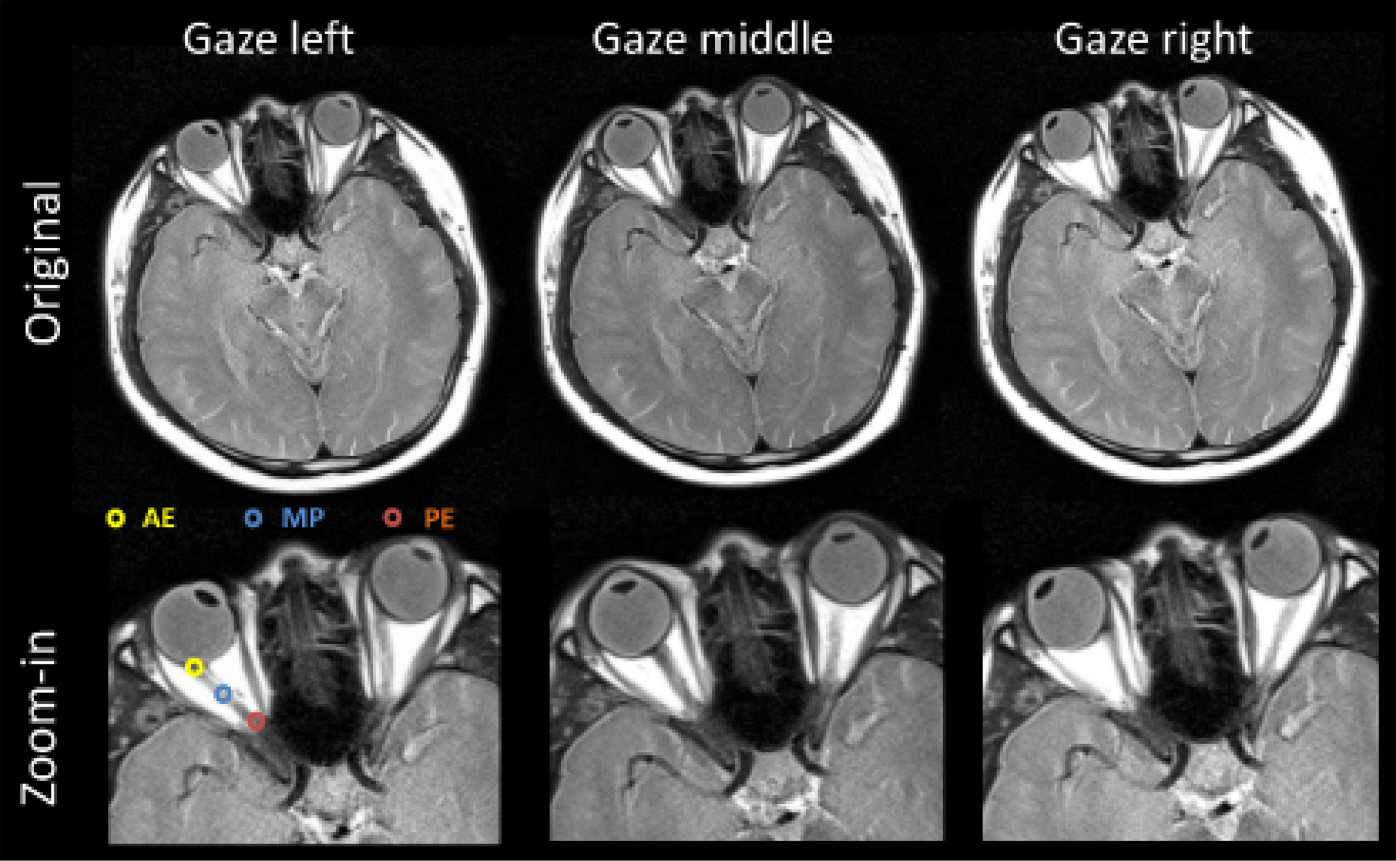
Representative axial view images obtained from subject S1 (29-year-old male). Optic nerve structure and measurement points are shown as: the anterior end (AE) of the optic nerve connects to the globe, the posterior end (PE) connects to the optic canal, and the middle point (MP) is the midpoint of the optic nerve. It is quite evident that the anterior ends of both optic nerves move quite substantially in the opposite direction of the motion of the eyes (lens), as compared to their locations when the subject is looking straight forward (gaze middle).

**Figure 2:**
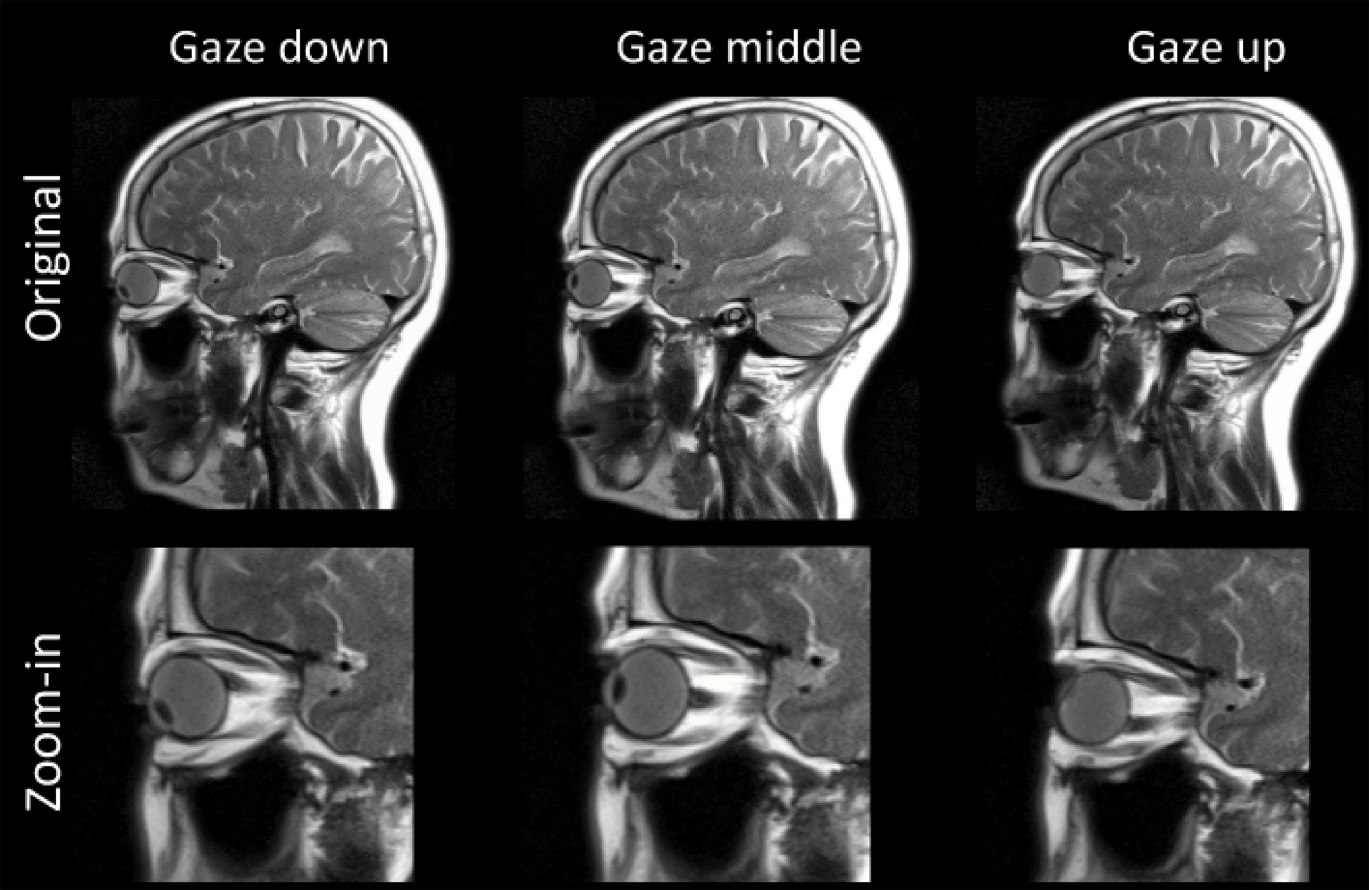
Representative images obtained from subject S4 (65-year-old female) for vertical movement of the eyes. Similarly, the anterior ends of both optic nerves move in the opposite direction of the vertical motion of the eyes (lens).

A representative case showing optic nerve movement in the left-right (L-R, axial plane) and superior-inferior (S-I, sagittal plane) directions is displayed in **Figures 1** and **2**. The middle images demonstrate the neutral position with the subject looking straight forward, while the others show the subject gazing in different directions. It is evident that the optic nerves move in the opposite direction relative to the lens. When the subject’s gaze was guided to the left, the optic nerves moved to the right, whereas when the subject was instructed to gaze upwards, the optic nerves moved downwards. Additionally, regardless of eye movement, the posterior end of the optic nerve, where it exits the optic canal, demonstrated minimal movement. Overall, the anterior end had a larger magnitude of displacement relative to the posterior end.

### Motion Assessment

**Figure 3:**
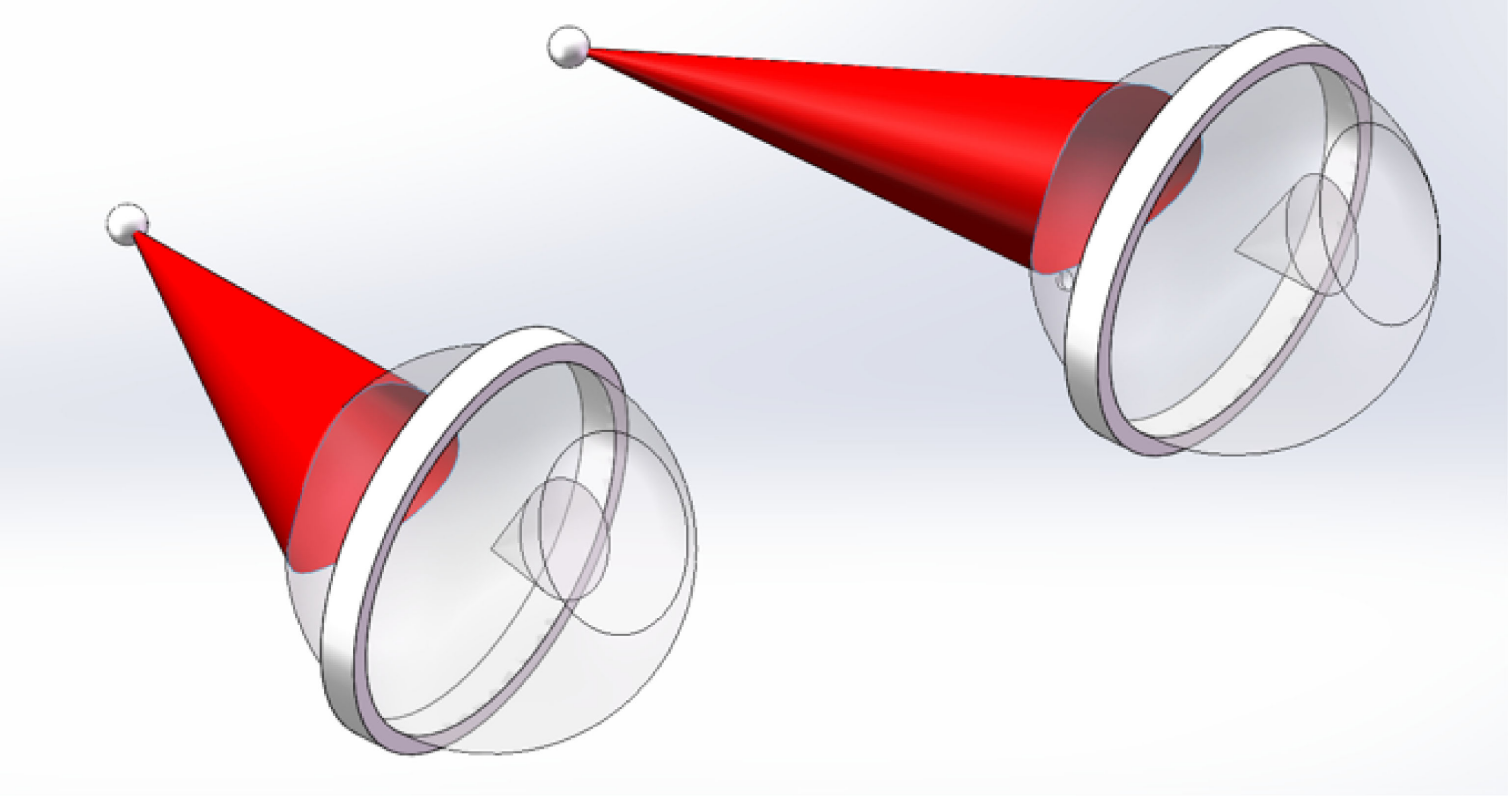
Images generated by Solidworks (Dassault Systèmes, Velizy-Villacoublay), showing the range of motion (red) for the optic nerves based on measurement of anatomy gathered from MRI of subject S1.

Movement was assessed *via* both horizontal and vertical movement. As shown in **Table 1**, the anterior end (AE) moved in the range of centimeters horizontally, with an average of 10.4 ± 2.2 mm and 11.1 ± 1.4 mm for the left and right eyes, respectively. Conversely, the posterior end (PE) had minimal movement, being within 1 mm. The middle point (MP) movement is equivalent for both eyes and is about half of the AE movement, with 5.1 ± 1.3 mm both the left and right eyes. The same motion pattern was observed regarding vertical movement. The anterior end moved with an average distance of 9.3 ± 0.8 mm going up/down, and the posterior end connecting to the optic canal showed minimal motion. From these results, it can be postulated that the optic nerve rotates around the PE, where it exits the optic canal, and moves within a conical shape, as shown in **Figure 3**.

**Table 1:** Horizontal movement of the optic nerves.

	Horizontal Movement						Vertical Movement		
	Right Eye			Left Eye			Right Eye		
	L*->C*	C->R*	L->R	L->C	C->R	L->R	U*->C	C->D	U->D
Anterior(mm)	4.9±0.8	6.2H.3	11.1+1.4	5.9±0.7	4.6±1.3	10.4±2.2	5.311.7	4.212.1	9.310.8
Middle(mm)	2.6+0.5	3.0±0.6	5.2±0.6	3.2±0.3	2.1±0.8	5.111.3	3.110.9	2.1+1.1	5.110.8
Posterior(mm)	0.7+0.3	0.5±0.2	0.8±0.3	0.3±0.4	0.3±0.2	0.410.2	0.4+0.3	0.4+0.3	0.5+0.3

*denotes five directions: L, Left; C, Center; R, Right; U, Upwards; D, Downwards.

### Repeatability:

Horizontal movement was tested in the three younger subjects (S1-S3). The mean differences were 1.1 mm for left eye and 1.2 mm for the right eye while the maximal differences for the left and right eyes were 2.7 and 3.8 mm respectively.

### Dosimetric Impact

Three clinical cases were selected to evaluate the dosimetric impact of possible optic nerve motion. Two cases (Patients #1 and 2) had optic nerves in close proximity to the target volumes, while a third case (Patient #3) had an optic nerve sheath meningioma where the nerve was part of the target volume itself. A conical shape with fixed PE and a 5 mm expansion for the AE was used to create a motion-inclusive planning optic nerve margin for all cases to evaluate the dosimetric impact further.

**Figure 4:**
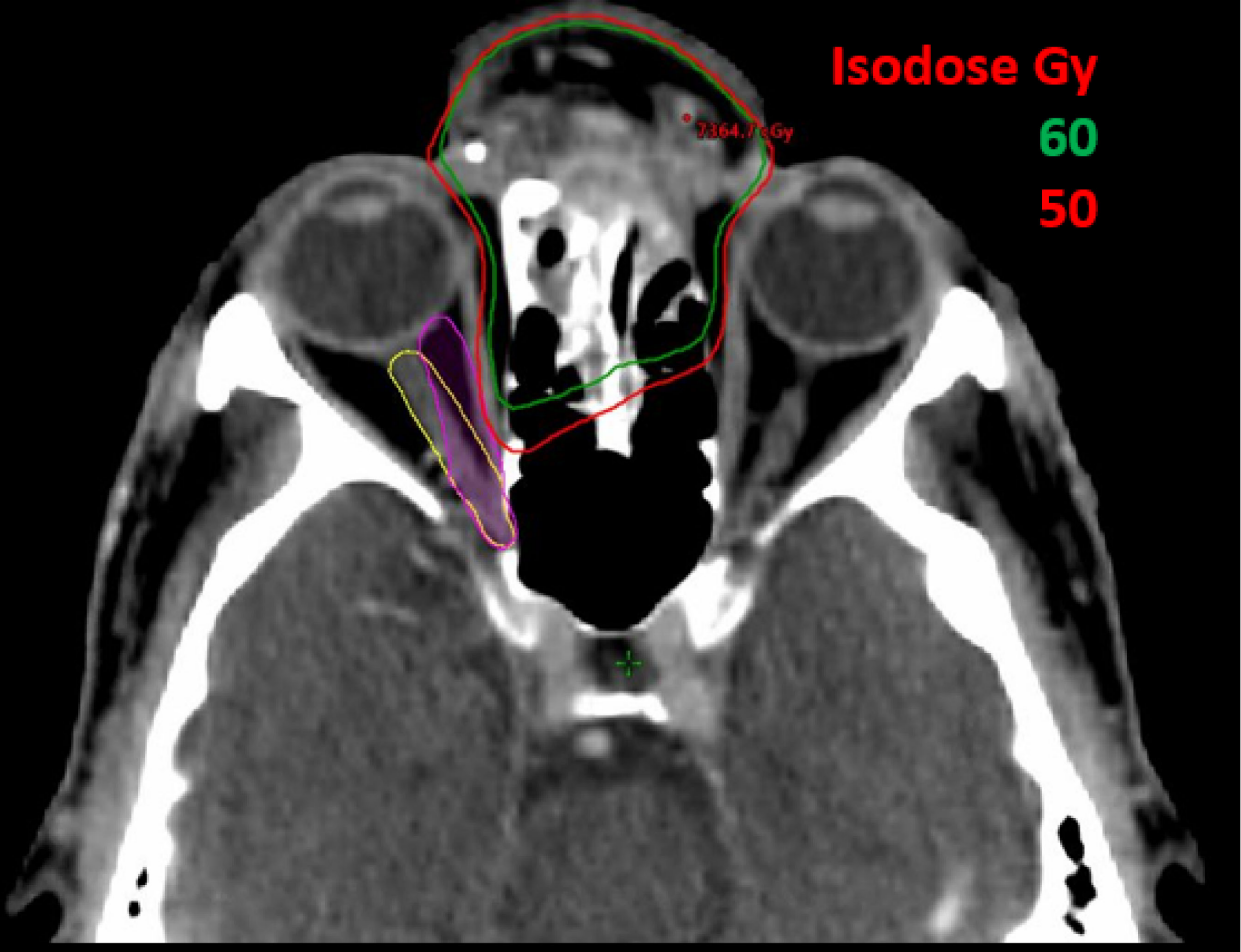
A 61-year-old male patient with skin cancer who was previously treated on Truebeam (Varian Medical Systems, Palo Alto, CA) for 66 Gy in 33 fractions. Original contour for optic nerve is shown in yellow and optic nerve with motion in worst scenario is shown in purple contour.

Patient #1, as shown in **Figure 4**, was diagnosed with a meningioma that treated with two partial arcs to a total dose of 6600 cGy in 33 fractions. In the original treatment plan, a conventional 3 mm expansion was used to create the optic nerve PRV. According to the latest QUANTEC recommendation for conventional fractionation (6), the estimated risk of toxicity for the optic apparatus is < 3% when the maximum point dose, D_max_ is less than 55Gy, 3%-7% if D_max_ is within 55-60Gy, and 7-20% when D_max_ is larger than 60Gy. In the patient’s original plan the D_max_ of the optic nerve is approximately 52 Gy (**Table 2**), which is within the tolerance limit; however, with the consideration of possible optic nerve movement, the D_max_ could have increased to 56 Gy, falling into the range of increased risk (3-7%) for developing optic neuropathy (RION).

**Table 2:** Changes of maximal doses due to potential movement of optic nerves.

	D_max_ (Gy)	D_0.035cc_ (Gy)	D_0.2cc_ (Gy)	D_0.5cc_ (Gy)
Patient #1	52.0→55.7	50.0→51.6	47.1→49.4	42.9→45.3
Patient #2	26.9→31.6	25.6→26.3	22.9→23.4	13.4→12.8*

Values before and after symbol → are original doses and doses after potential movement of optic nerve. * decrease of D0.5cc is because the total volume of the optic nerve is 0.7cc. Even though the maximal dose increases, the minimal dose covering the 0.5cc volume decreases.

**Figure 5:**
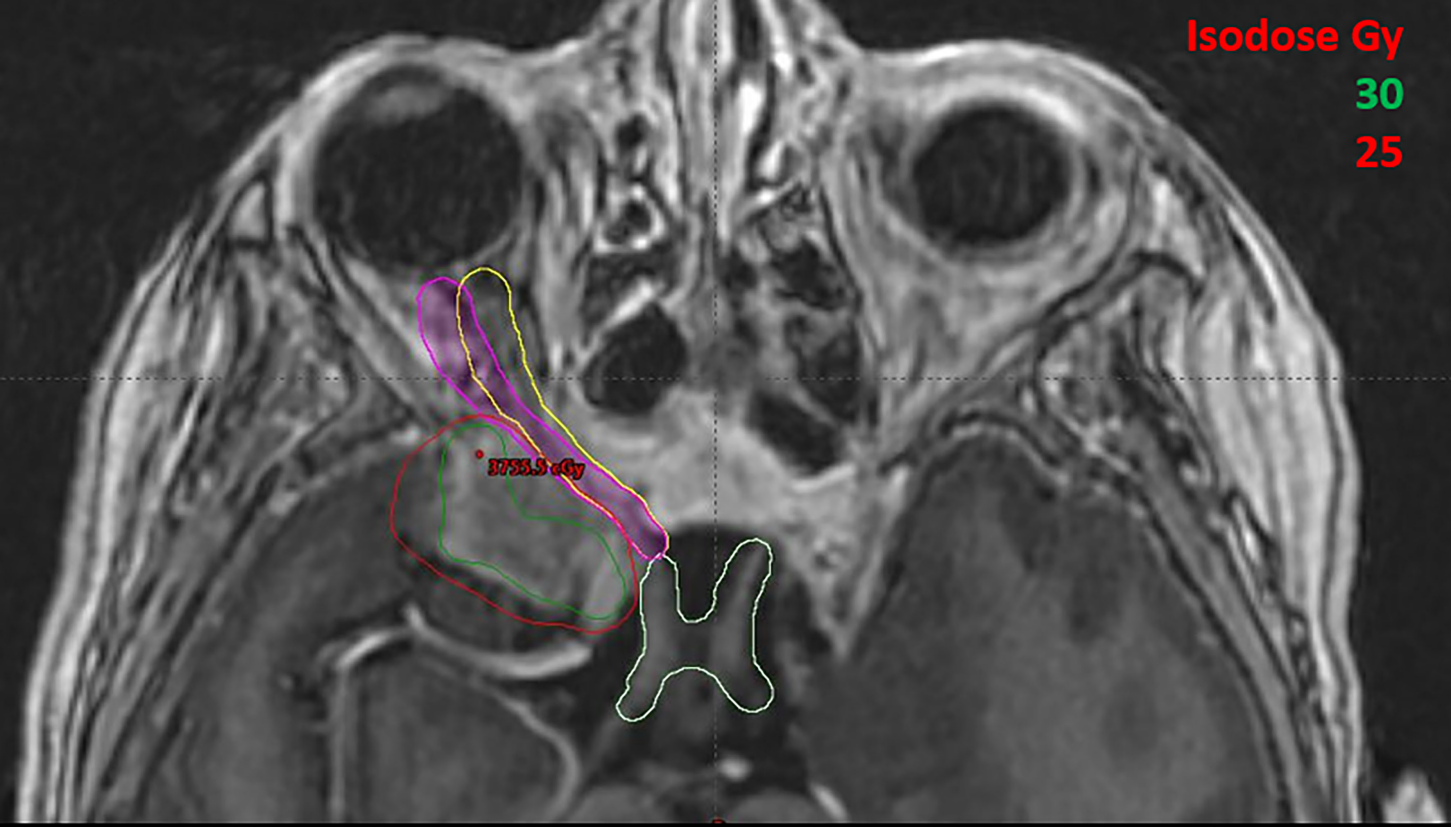
A female patient with a perioptic pituitary adenoma treated with hypofractionated SRS for 2500 cGy in 5 fractions. Original contour for optic nerve is shown in yellow and optic nerve with motion in worst scenario is shown in purple contour.

Patient #2, as shown in **Figure 5**, was diagnosed with a peri-optic pituitary adenoma treated with hypofractionated SRS to a total dose of 2500 cGy in 5 fractions. When utilizing the motion-inclusive margin, the maximum point dose D_0.035 cc_ (the dose covering the 0.035cc volume receiving highest dose), as shown in **Table 2**, increased from 24.4 Gy (clinically acceptable) to 25.2 Gy, which is above the dose constraint of 25 Gy, in accordance with AAPM TG-101 (7). The D_0.2cc_ (the dose covering the 0.2cc volume receiving highest dose) increased from 22.9 Gy to 23.4 Gy, which also exceeded the recommended dose constraints (≤23Gy) as per protocol (7).

**Figure 6:**
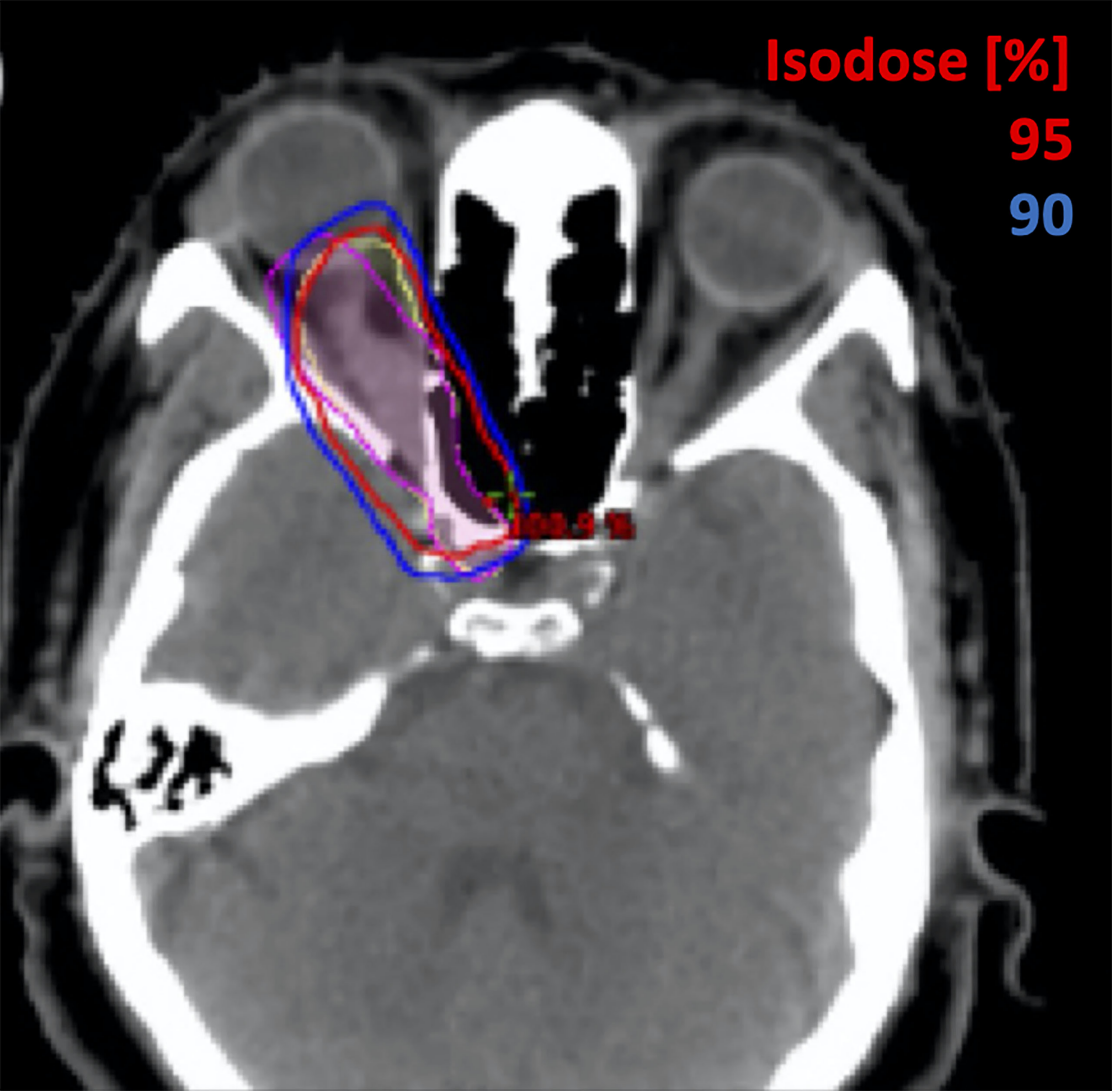
A 51-year-old female with a benign meningioma treated on the Mevion S250 Proton Therapy System (Mevion Medical Systems, Littleton, MA) for 52.2 Gy in 29 fractions. Original contours for GTV and PTV are shown in yellow and GTV and PTV moving with optic nerve in worst scenario are shown in purple contours.

Patient #3, as shown in **Figure 6**, was diagnosed with an optic nerve sheath meningioma with a large portion of the optic nerve itself being targeted with proton beam therapy to a total relative biologically effective (RBE) dose of 5220cGy in 29 fractions. The PTV percent volume that received at least 95% of the prescription dose (D_95_, yellow) with conventional 3mm expansion from the GTV was 95% of the prescription dose in the treated plan; however if the motion-inclusive expansion PTV (purple) was utilized, the D_95_ target coverage actually dropped to 92%, as shown in **Figure 6**. The minimum dose, D_min_, to the gross tumor tablevolume (GTV) also dropped from 95% of the prescription dose in the treated plan to 90% with the motion-inclusive expansion as shown in **Figure 6**.

## Discussion

The purpose of this study was to identify the extent of optic nerve motion using MRI and to further evaluate the dosimetric impact on perioptic lesions that have undergone radiation treatment. Optic nerve motion was found to be in the opposite direction of globe motion and followed a nearly conical shape. The displacement of the PEs of the optic nerves, where they adjoin the optic canal, was minimal (within 1 mm) in all subjects, yet the displacement of the AEs of the nerves, where they connect to the globes, could be over 10 mm. A simple rigid motion model was applied to three clinical scenarios to evaluate the dosimetric effects of optic nerve movement based on the finding of this study. In these three cases, non-negligible dosimetric changes were observed.

A few studies have investigated optic nerve motion previously. Clarke et al. (8) demonstrated that by having patients look at different sides the optic nerves could move up to 6 mm as compared to the neutral position, based on CBCT images of four patients. In a study investigating optimal MRI sequence design for optic nerve disease by Moodley et al. (4), it was found that the mean total distance that the optic nerves travel during eye movement was 11.8 mm; however, none of these prior studies systematically evaluated the movement patterns for the optic nerves in multiple directions, nor did they further assess the dosimetric impact of these movements.

Although the absolute risks of RION remain relatively low in most patients, the risk for toxicity increases exponentially as therapeutic dose increases. This is especially true in patients with peri-optic lesions such as meningiomas, pituitary adenomas etc., where hypofractionated SRS can be the definitive treatment. Due to sharp dose falloff required near critical structures with SRS, even small uncertainties in position could have a sizeable effect on the radiation dose delivered. The risk of RION can be further increased in patients who have undergone prior radiation treatments to the same area, where avoiding excessive dose to the optic nerve is crucial. For SRS, most clinical sites use either no margin or 1 mm margins for the optic nerve contours/PRV. For conventional external beam treatment, a 3 to 5 mm margin is generally used, pending image guidance and treatment technique. The results obtained from this study demonstrated that the displacement of the optic nerves as patients change the direction of their gaze can exceed these defined margins, and this may lead to insufficient dosing to the target volume if the tumor is of the optic nerve (i.e., optic sheath meningioma), or excessive dosage to the organs at risk (i.e., optic nerve itself) if the PTV is adjacent to the nerve. One may argue that for conventionally fractionated external beam radiation therapy, treatment is divided into multiple fractions over a long course. The eyeball movement is likely be averaged out in all directions. However, for single-fraction SRS or hypofractionated SRT, the optic nerve motion could result in a higher impact, especially when patients are treated with high-dose-rate beams (e.g. 1400MU/min for 6FFF beam). Additionally, majority of patients treated in radiation oncology are simulated with CT images, which only take a few seconds. If the patient is simulated with optic nerves at non-neutral positions the dosimetric estimation regarding the nerves itself could be inaccurate since the treatment plan was purely generated based on that image set. A possible solution to this could be that patients may need to be counseled to look straight ahead (or in a pre-determined direction based on target location) or to utilize eye-tracking if deemed necessary (9, 10). Furthermore, during treatment, it may be beneficial to guide the patient to look towards the desired optimal side/direction, in order to minimize the radiation dose to the optic nerve. Nevertheless, data on how far and frequently the patients move their eyes during the entire course of radiation treatment and how stable the optic nerve can stay at neutral position with/without couching, although beyond the scope of the current study, warrants larger-scale clinical investigations.

This study has a few limitations. First, to shorten the imaging acquisition time for improved patient comfort, two-dimensional imaging sequences with relatively thick slices (3mm) were used. Even though the in-plane resolution is relatively high (0.7 mm), the thicker slices could still lead to uncertainty in the measured range of movement. Given the imaging parameters, this uncertainty should not exceed 1-2 mm, which does not affect the conclusion of this study. In addition, a rigid movement model was assumed throughout the course of the study. The optic nerve, being an organ consisting of soft tissue, deforms with motion. Based on imaging results, the anterior portion of the optic nerves, which are close to the posterior walls of the globes, followed the rotation of the globes and bent towards their posterior aspects. This slightly reduces the actual movement of the optic nerves following the motion of the globes. The rigid movement model used in this study serves as a conservative estimate for the range of motion. It also shows that optic nerves maintain linear shapes following movement. This is consistent with the findings that displacement of the middle points of the optic nerves is always close to half of the displacement values measured for the anterior portions. Therefore, a rigid movement model, which may overestimate the movement slightly, should still provide a very reasonable and relatively accurate estimation of the range of motion. Finally, the clinical impact of the dosimetric changes found based on the motion-inclusive model warrants more investigation. A prospective study with larger cohort of patients would better elucidate the overall impact on reducing the incidence of RION or improving tumor control, especially in stereotactic radiation therapy with reduced margins of the OARs and/or targets. Future work with accumulation of relevant cases with thinner image slices and longitudinal follow-up will be needed.

## Conclusion

In this exploratory study, the optic nerves were found to follow a nearly conical shape relatively to the motion of the eyes. The range of motion of the anterior portions of the optic nerves was on the order of centimeters, which exceeds the current considerations in the field of clinical radiation oncology. Special attention may be needed for radiation simulation, treatment planning, and treatment monitoring to avoid excessive dosage to the optic nerves, or to ensure sufficient coverage of the target on the optic nerves, depending on varying clinical scenarios.

## Data Availability Statement

The raw data supporting the conclusions of this article will be made available by the authors, without undue reservation.

## Ethics Statement

The studies involving human participants were reviewed and approved by the University of Virginia. The patients/participants provided their written informed consent to participate in this study.

## Author Contributions

KQ set up the MR protocols, analyzed the data, and wrote the manuscript. KN and AC designed the study, provided patient data, evaluated treatment plan, and revised the manuscript. JS evaluated the MR protocols, supervised the MR studies. JW provided patient data and evaluated the treatment plan. BL prepared **Figures 2**–**6**, assisted KQ to analyze the data, and revised the manuscript. XF scanned the healthy volunteers on MR scanner and assisted KQ to collect imaging data. TC, YZ, JZ, and XW provided patient data and helped KQ to generate comparison treatment plan. QC analyzed the MRI data and assisted in manuscript writing SP assisted in manuscript writing and data analysis. SK provided patient data and evaluated treatment plan. All authors contributed to the article and approved the submitted version.

## Conflict of Interest

The authors declare that the research was conducted in the absence of any commercial or financial relationships that could be construed as a potential conflict of interest.

## Publisher’s Note

All claims expressed in this article are solely those of the authors and do not necessarily represent those of their affiliated organizations, or those of the publisher, the editors and the reviewers. Any product that may be evaluated in this article, or claim that may be made by its manufacturer, is not guaranteed or endorsed by the publisher.
